# Supplementary material for: CRISPRi-seq in Haemophilus influenzae reveals genome-wide and medium-specific growth determinants
Source: PLoS Pathog. 2025 Oct 31;21(10):e1013650. doi: 10.1371/journal.ppat.1013650 (PMC12591398; doi:10.1371/journal.ppat.1013650)
Supplement: S1 Text — Table A. Bacterial strains used in this study. Table B. Composition of CDM and mCDM media used in this study. Table C. Plasmids used in this study. Table D. Primers used in this study. Fig A. Dynamic range of the aTc-inducible CRISPRi system in H. influenzae. (A) Growth of H. influenzae WT (triangles) and dcas9 (circles) strains in sBHI. (B) Ptet responsiveness experiment: dcas9 strains carrying no (-) or sgRNAs targeting the sspA, fabH or glyA genes were grown for 12 h at 37°C in 10 mL sBHI with shaking (120 r.p.m). Next, cultures were diluted in fresh sBHI to OD600 = 0.01 and transferred to a 96-wells plate with a range of aTc concentrations from 0.25 to 50 ng/mL. The color-coded shadow area represents the standard deviation to the mean (solid line). Fig B. Frequency histogram of the E. coli and H. influenzae sgRNA library distribution. sgRNA library distribution in E. coli (A) and in H. influenzae (B). Fig C. sgRNA content variation during CRISPRi screen in sBHI and CDM. (A) Principal component analysis (PCA) shows the disparity between replicates, induction (+ or – aTc), media (sBHI, CDM) and timepoints (7, 14 and 21 generations). + signs represent samples where CRISPRi was activated by aTc; - signs show samples where CRISPRi was uninduced. Blue and red signs represent CRISPRi libraries grown in sBHI and CDM, respectively. Increasing the induction time explains most of the variance in sample strain composition (91%). (B) Clustered heatmap of sgRNA content between replicates, induction (+ or – aTc), media (sBHI, CDM) and timepoints (7, 14 and 21 generations). The normalized read count is expressed in a gradient logarithmic scale. The horizontal axis displays the 1,773 sgRNAs designed for the RdKW20 CRISPRi library, while the vertical axis shows the 48 different samples. Fig D. CRISPRi-based analysis of H. influenzae specific gene fitness in sBHI. dcas9 derivative strains expressing infC, rpsL, ispE, metK, rpoC, lpxB, parE, rsxA, rpsT and mepA sgRNAs were grown in [file ppat.1013650.s008.docx]

**CRISPRi-seq in *Haemophilus influenzae* reveals genome-wide and medium-specific growth determinants by** Celia Gil-Campillo et al.

Supporting Information files:

**Table A.** Bacterial strains used in this study.

| **Strain** | **Description** | **Source** |
| --- | --- | --- |
| ***E. coli*** |  |  |
| TOP10 | Cloning strain. F-*mcrA*Δ(*mrr-hsd*RMS-*mcr*BC) Φ80*lac*ZΔM15 Δ*lac*X74 *rec*A1 *ara*D139 Δ(*ara,leu*)7697 *gal*U*gal*K*rps*L (Str^R^) *end*A1 *nup*G | Fisher Scientific |
| StbI3 | Cloning strain. F^-^*mcr*B *mrrhsd*S20(r_B_^-^,m_B_) *rec*A13 *sup*E44 *ara*14 *gal*K2 *lac*Y1 *pro*A2 *rps*L20(Str^R^) *xyl*-5 λ^-^*leumtl*-1 | Fisher Scientific |
| StbI3 pPEPzHi-sgRNA clone/P1419 | StbI3 derivative, containing pPEPzHi-sgRNA plasmid (Hi061.1upstream-Read1-P3-*BsmB*I-*mCherry*-*BsmB*I-dCas9handle-ter-Read2-*spec*-Hi061.1downstream); Spec^R^ | This study |
| ***S. pneumoniae*** |  |  |
| VL3468 | D39, *tetM*-P*_F6_*-*tetR*-P*_tet_*-*dCas9* cassette with a *tetR* gene and a tet-inducible *dCas9* (D10A, H840A) integrated at the CEP *locus*; Tet^R^ | Lab collection |
| ***H. influenzae*** |  |  |
| RdKW20 | Laboratory strain, capsule-deficient serotype d | (1) |
| NTHi375 | Clinical isolate, otitis media origin | (2) |
| 86-028NP | Clinical isolate, otitis media origin | (3) |
| R2866 | Clinical isolate, blood isolate | (4) |
| RdKW20-dCas9/P1515 | RdKW20 derivative containing a *tetR*-P_tet_-*dCas9* cassette chromosomally integrated in the *xylB*-*rfaD* region; Erm^R^ | This study |
| RdKW20-dCas9 P3-sgRNA*^sspA^* (3)/P1518 | RdKW20-dCas9/P1515 derivative containing a P3-*sspA* sgRNA cassette chromosomally integrated in the Hi0601.1 region, clone 3; Erm^R^, Spec^R^ | This study |
| RdKW20-dCas9 P3-sgRNA*^fabH^* (4)/P1521 | RdKW20-dCas9/P1515 derivative containing a P3-*fabH* sgRNA cassette chromosomally integrated in the Hi0601.1 region, clone 45; Erm^R^, Spec^R^ | This study |
| RdKW20-dCas9 P3-sgRNA*^glyA^* (2)/P1523 | RdKW20-dCas9/P1515 derivative containing a P3-*glyA* sgRNA cassette chromosomally integrated in the Hi0601.1 region, clone 2; Erm^R^, Spec^R^ | This study |
| RdKW20-dCas9 P3-sgRNA^HI_t06^ (5)/P1587 | RdKW20-dCas9/P1515 derivative containing a P3-HI_t06 sgRNA cassette chromosomally integrated in the Hi0601.1 region, clone 5; Erm^R^, Spec^R^ | This study |
| RdKW20-dCas9 P3-sgRNA^dnaE^(5)/P1606 | RdKW20-dCas9/P1515 derivative containing a P3-*dnaE* sgRNA cassette chromosomally integrated in the Hi0601.1 region, clone 5; Erm^R^, Spec^R^ | This study |
| RdKW20-dCas9 P3-sgRNA^rpoH^(1)/P1572 | RdKW20-dCas9/P1515 derivative a containing a P3-*rpoH* sgRNA cassette chromosomally integrated in the Hi0601.1 region, clone 1; Erm^R^, Spec^R^ | This study |
| RdKW20-dCas9 P3-sgRNA^rplO^(2)/P1584 | RdKW20-dCas9/P1515 derivative containing a P3-*rplO* sgRNA cassette chromosomally integrated in the Hi0601.1 region, clone 2; Erm^R^, Spec^R^ | This study |
| RdKW20-dCas9 P3-sgRNA^infC^(1)/P1575 | RdKW20-dCas9/P1515 derivative containing a P3-*infC* sgRNA cassette chromosomally integrated in the Hi0601.1 region, clone 1; Erm^R^, Spec^R^ | This study |
| RdKW20-dCas9 P3-sgRNA^dnaA^(1)/P1602 | RdKW20-dCas9/P1515 derivative containing a P3-*dnaA* sgRNA cassette chromosomally integrated in the Hi0601.1 region, clone 1; Erm^R^, Spec^R^ | This study |
| RdKW20-dCas9 P3-sgRNA^ispE^(1)/P1662 | RdKW20-dCas9/P1515 derivative containing a P3-*ispE* sgRNA cassette chromosomallyintegrated in the Hi0601.1 region, clone 1; Erm^R^, Spec^R^ | This study |
| RdKW20-dCas9 P3-sgRNA^metK^(1)/P1578 | RdKW20-dCas9/P1515 derivative containing a P3-*metK*sgRNA cassette chromosomally integrated in the Hi0601.1 region, clone 1; Erm^R^, Spec^R^ | This study |
| RdKW20-dCas9 P3-sgRNA^rpoC^(4)/P1570 | RdKW20-dCas9/P1515 derivative containing a P3-*rpoC* sgRNA cassette chromosomally integrated in the Hi0601.1 region, clone 4; Erm^R^, Spec^R^ | This study |
| RdKW20-dCas9 P3-sgRNA^lpxB^(2)/P1639 | RdKW20-dCas9/P1515 derivative containing a P3-*lpxB* sgRNA cassette chromosomally integrated in the Hi0601.1 region, clone 2; Erm^R^, Spec^R^ | This study |
| RdKW20-dCas9 P3-sgRNA^parE^(1)/P1653 | RdKW20-dCas9/P1515 derivative containing a P3-*parE* sgRNA cassette chromosomally integrated in the Hi0601.1 region, clone 1; Erm^R^, Spec^R^ | This study |
| RdKW20-dCas9 P3-sgRNA^rsxA^(1)/P1644 | RdKW20-dCas9/P1515 derivative containing a P3-*rsxA* sgRNA cassette chromosomally integrated in the Hi0601.1 region, clone 1; Erm^R^, Spec^R^ | This study |
| RdKW20-dCas9 P3-sgRNA^guaB^(7)/P1630 | RdKW20-dCas9/P1515 derivative containing a P3-*guaB* sgRNA cassette chromosomally integrated in the Hi0601.1 region, clone 7; Erm^R^, Spec^R^ | This study |
| RdKW20-dCas9 P3-sgRNA^ilvE^(5)/P1624 | RdKW20-dCas9/P1515 derivative containing a P3-*ilvE* sgRNA cassette chromosomally integrated in the Hi0601.1 region, clone 5; Erm^R^, Spec^R^ | This study |
| RdKW20-dCas9 P3-sgRNA^serA^(1)/P1656 | RdKW20-dCas9/P1515 derivative containing a P3-*serA* sgRNA cassette chromosomally integrated in the Hi0601.1 region, clone 1; Erm^R^, Spec^R^ | This study |
| RdKW20-dCas9 P3-sgRNA^rpsT^(4)/P1608 | RdKW20-dCas9/P1515 derivative containing a P3-*rpsT* sgRNA cassette chromosomally integrated in the Hi0601.1 region, clone 4; Erm^R^, Spec^R^ | This study |
| RdKW20-dCas9 P3-sgRNA^mepA^(7)/P1591 | RdKW20-dCas9/P1515 derivative containing a P3-*mepA* sgRNA cassette chromosomally integrated in the Hi0601.1 region, clone 7; Erm^R^, Spec^R^ | This study |
| RdKW20-dCas9 P3-sgRNA^ftsZ^(1)/P1680 | RdKW20-dCas9/P1515 derivative containing a P3-*ftsZ* sgRNA cassette chromosomally integrated in the Hi0601.1 region, clone 1; Erm^R^, Spec^R^ | This study |
| RdKW20-dCas9 P3-sgRNA^rpsL^(1)/P1650 | RdKW20-dCas9/P1515 derivative containing a P3-*rpsL* sgRNA cassette chromosomally integrated in the Hi0601.1 region, clone 1; Erm^R^, Spec^R^ | This study |
| RdKW20 ∆*serA*/P1698 | *serA*::*ermC,* Erm^R^ cassette inactivation cassette | This study |

**Table B.** Composition of CDM and mCDM media used in this study.

| **Components** | **mCDM** | **CDM** (5, 6) |
| --- | --- | --- |
|  | **Concentration (mM)** | **Concentration (mM)** |
| HEPES 1M | 24.7 | 24.7 |
| Uracil (2 mg/mL) | 0.76 | 0.76 |
| Inosine (20 mg/mL) | 6.33 | 6.33 |
| NAD (1 mg/mL) | 0.00146 | 0.00146 |
| Hemin (1 mg/mL) | 0.0153 | 0.0153 |
| Glucose | 10 | 10 |
| Sodium pyruvate | 0 | 0.085 |
| Choline chloride | 0.02 | 0.02 |
| D-Calcium pantothenate | 0.00052 | 0.00052 |
| Thiamine hydrochloride | 0.003 | 0.003 |
| Biotin | 0 | 8.1 x 10^-4^ |
| Folic Acid | 0 | 0.0023 |
| Niacinamide | 0 | 0.0082 |
| Para-Aminobenzoic Acid | 0 | 0.0072 |
| Pyridoxine hydrochloride | 0 | 0.0049 |
| Riboflavin | 0 | 5.3 x 10^-4^ |
| Vitamin B12 | 0 | 3.7 x 10^-6^ |
| i-Inositol | 0 | 0.19 |
| Phenol Red | 0 | 0.013 |
| Calcium nitrate (Ca(NO3)2. 4H_2_O) | 0.42 | 0.42 |
| Magnesium sulfate (MgSO4) (anhyd.) | 0.407 | 0.407 |
| Potassium chloride (KCl) | 5.33 | 5.33 |
| Glutathione (reduced) | 0.0032 | 0.0032 |
| Sodium bicarbonate (NaHCO_3_) | 23.81 | 23.81 |
| Sodium chloride (NaCl) | 103.45 | 103.45 |
| Sodium phosphate dibasic (Na_2_HPO4-7H_2_O) | 5.64 | 5.64 |
| Glycine | 0.13 | 0.13 |
| L-Arginine | 1.15 | 1.15 |
| L-Asparagine | 0.38 | 0.38 |
| L-Aspartic acid | 0.15 | 0.15 |
| L-Cystine 2HCl | 0.21 | 0.21 |
| L-Glutamic Acid | 0.14 | 0.14 |
| L-Glutamine | 2.05 | 2.05 |
| L-Histidine | 0.10 | 0.10 |
| L-Isoleucine | 0.38 | 0.38 |
| L-Leucine | 0.38 | 0.38 |
| L-Lysine hydrochloride | 0.27 | 0.27 |
| L-Methionine | 0.10 | 0.10 |
| L-Phenylalanine | 0.09 | 0.09 |
| L-Proline | 0.17 | 0.17 |
| L-Threonine | 0.17 | 0.17 |
| L-Tyrosine disodium salt dihydrate | 0.11 | 0.11 |
| L-Valine | 0.17 | 0.17 |
| L-Tryptophan | 0 | 0.025 |
| L-Serine | 0 | 0.29 |

**Table C.** Plasmids used in this study.

| **Plasmid** | **Description of cassette** | **Source** |
| --- | --- | --- |
| pPEPZ-sgRNAclone | ZIP-*spec*-P3-*mCherry*-dCas9handle-terminator-ZIP; Spec^R^. Stored in strain P1394 | (7) |
| pPEPZHi-*mCherry* | Hi0601.1upstream-Read1-P3-*BsmB*I-*mCherry*-*BsmB*I-dCas9handle-ter-Read2-*spec*-Hi0601.1downstream; Spec^R^. Stored in strain P1419 | This study |
| pPEPZHi-sgRNA*_fabH_* | Hi0601.1upstream-Read1-P3-*fabH*-dCas9handle-ter-Read2-*spec*-Hi0601.1downstream; Spec^R^. Stored in strain P1485 | This study |
| pPEPZHi-sgRNA*_glyA_* | Hi0601.1upstream-Read1-P3-*glyA*-dCas9handle-ter-Read2-*spec*-Hi0601.1downstream; Spec^R^. Stored in strain P1482 | This study |
| pPEPZHi-sgRNA*_sspA_* | Hi0601.1upstream-Read1-P3-*sspA*-dCas9handle-ter-Read2-*spec*-Hi0601.1downstream; Spec^R^. Stored in strain P1483 | This study |
| pPEPZHi-sgRNA_HI_t06_ | Hi0601.1upstream-Read1-P3-HI_t06-dCas9handle-ter-Read2-*spec*-Hi0601.1downstream; Spec^R^. Stored in strain P1684 | This study |
| pPEPZHi-sgRNA*_dnaE_* | Hi0601.1upstream-Read1-P3-*dnaE*-dCas9handle-ter-Read2-*spec*-Hi0601.1downstream; Spec^R^. Stored in strain P1537 | This study |
| pPEPZHi-sgRNA*_rpoH_* | Hi061.1upstream-Read1-P3-*rpoH*-dCas9handle-ter-Read2-*spec*-Hi061.1downstream; Spec^R^. Stored in strain P1535 | This study |
| pPEPZHi-sgRNA*_rplO_* | Hi0601.1upstream-Read1-P3-*rplO*-dCas9handle-ter-Read2-*spec*-Hi0601.1downstream; Spec^R^. Stored in strain P1683 | This study |
| pPEPZHi-sgRNA*_infC_* | Hi0601.1upstream-Read1-P3-*infC*-dCas9handle-ter-Read2-*spec*-Hi0601.1downstream; Spec^R^. Stored in strain P1541 | This study |
| pPEPZHi-sgRNA*_dnaA_* | Hi0601.1upstream-Read1-P3-*dnaA-*dCas9handle-ter-Read2-*spec*-Hi0601.1downstream; Spec^R^. Stored in strain P1536 | This study |
| pPEPZHi-sgRNA*_ispE_* | Hi0601.1upstream-Read1-P3-*ispE-*dCas9handle-ter-Read2-*spec*-Hi0601.1downstream; Spec^R^. Stored in strain P1601 | This study |
| pPEPZHi-sgRNA*_metK_* | Hi0601.1upstream-Read1-P3-*metK*-dCas9handle-ter-Read2-*spec*-Hi0601.1downstream; Spec^R^. Stored in strain P1540 | This study |
| pPEPZHi-sgRNA*_rpoC_* | Hi0601.1upstream-Read1-P3-*rpoC*-dCas9handle-ter-Read2-*spec*-Hi0601.1downstream; Spec^R^. Stored in strain P1534 | This study |
| pPEPZHi-sgRNA*_lpxB_* | Hi0601.1upstream-Read1-P3-*lpxB*-dCas9handle-ter-Read2-*spec*-Hi0601.1downstream; Spec^R^. Stored in strain P1557 | This study |
| pPEPZHi-sgRNA*_parE_* | Hi0601.1upstream-Read1-P3-*parE*-dCas9handle-ter-Read2-*spec*-Hi0601.1downstream; Spec^R^. Stored in strain P1562 | This study |
| pPEPZHi-sgRNA*_rsxA_* | Hi0601.1upstream-Read1-P3-*rsxA*-dCas9handle-ter-Read2-*spec*-Hi0601.1downstream; Spec^R^. Stored in strain P1559 | This study |
| pPEPZHi-sgRNA*_guaB_* | Hi0601.1upstream-Read1-P3-*guaB*-dCas9handle-ter-Read2-*spec*-Hi0601.1downstream; Spec^R^. Stored in strain P1554 | This study |
| pPEPZHi-sgRNA*_ilvE_* | Hi0601.1upstream-Read1-P3-*ilvE*-dCas9handle-ter-Read2-*spec*-Hi0601.1downstream; Spec^R^. Stored in strain P1551 | This study |
| pPEPZHi-sgRNA*_serA_* | Hi0601.1upstream-Read1-P3-*serA*-dCas9handle-ter-Read2-*spec*-Hi0601.1downstream; Spec^R^. Stored in strain P1593 | This study |
| pPEPZHi-sgRNA*_rpsT_* | Hi0601.1upstream-Read1-P3-*rpsT*-dCas9handle-ter-Read2-*spec*-Hi0601.1downstream; Spec^R^. Stored in strain P1539 | This study |
| pPEPZHi-sgRNA*_mepA_* | Hi0601.1upstream-Read1-P3-*mepA*-dCas9handle-ter-Read2-*spec*-Hi0601.1downstream; Spec^R^. Stored in strain P1542 | This study |
| pPEPZHi-sgRNA*_ftsZ_* | Hi0601.1upstream-Read1-P3-*ftsZ*-dCas9handle-ter-Read2-*spec*-Hi0601.1downstream; Spec^R^. Stored in strain P1600 | This study |
| pPEPZHi-sgRNA*_rpsL_* | Hi0601.1upstream-Read1-P3-*rpsL*-dCas9handle-ter-Read2-*spec*-Hi0601.1downstream; Spec^R^. Stored in strain P1561 | This study |
| pJET1.2-*serA* | pJET1.2 derivative containing a 1,240 bp DNA fragment carrying the *serA* gene. Stored in strain P1259 | This study |
| pJET1.2-*serA*::*spec* | pJET1.2-*serA* derivative containing a 2,410 bp DNA fragment carrying a *serA::spec* disruption cassette. Stored in strain P1260 | This study |
|  |  |  |

**Table D.** Primers used in this study.

| **Primer name/number** | **Primer sequence (5’-3’)** | **Purpose** |
| --- | --- | --- |
| F_*xylB*/2469 | TGGCTAACAGGAAAATTCTGCACT | CRISPRi platform |
| R_*Aar*I_xylB-ery/2470 | TATTACACCTGCACTAACCCCTATAGCTTATCGGTATAGGGG | CRISPRi platform |
| F_*Aar*I_ery/2471 | TATTACACCTGCACTAGGGTACCGAGCTCGAATTCG | CRISPRi platform |
| R_*Aar*I_ery/2472 | TATTACACCTGCACTATGGAGCTCGAATTCGGCTTCATG | CRISPRi platform |
| F_*Aar*I_tetR/2473 | TATTACACCTGCACTATCCATGCGGCAAAGCACTCAAAAG | CRISPRi platform |
| R_*Aar*I_dCas9/2474 | TATTACACCTGCACTACCTGTACAGTTACCATACGTAGGTG | CRISPRi platform |
| F_*Aar*I_*rfaD*/2475 | TATTACACCTGCACTACAGGATTTACTACATTAATAATTTATTTTCTATTCAACCACGCC | CRISPRi platform |
| R_*rfaD*/2476 | TGATTATCGTAACAGGTGGCGC | CRISPRi platform |
| F_*Aar*I-Hi0601up/ 2477 | TATTACACCTGCACGTTCCCAAGACGAGTTATCAAAGAATT | CRISPRi platform |
| R_*Aar*I-Hi0601up/ 2478 | TATTACACCTGCACGTTGCGACAATCAAACCAATAAACCC | CRISPRi platform |
| R_*Aar*I-sgRNA/2480 | TATTACACCTGCACGTAAAAATATGATTGCCCTCTTGTTC | CRISPRi platform |
| R_*Aar*I_Hi0601dn/ 2482 | TATTACACCTGCACGTGGTGACTAAATCTCATCAAAGCAG | CRISPRi platform |
| F_*Aar*I-oUC18/2483 | TATTACACCTGCACGTCACCCCGTAGAAAAGATC | CRISPRi platform |
| R_AarI-oUC18/2484 | TATTACACCTGCACGTGGGATAACGCAGGAAAGAAC | CRISPRi platform |
| F_AarI-specHi/2485 | TATTACACCTGCACGTTTTTGTCGACGGTATCGATAAGC | CRISPRi platform |
| F_AarI-P3-sgRNA/2488 | TATTACACCTGCACGTCGCATTTTTCGCAATTCGGTCGAC | CRISPRi platform |
| R_AarI_specmut/  2517 | TATTACACCTGCACGTCAAGTGTTAAAGACCGCAA | CRISPRi platform |
| F_AarI_specmut/2518 | TATTACACCTGCACGTCTTGCAGTATCCAAAGAACC | CRISPRi platform |
| fabH_sgRNA_Fw/ 2530 | TATAGCTCGGCAGATAGCTACCGG | validation CRISPRi |
| fabH_sgRNA_Rv/2531 | AAACCCGGTAGCTATCTGCCGAGC | validation CRISPRi |
| glyA_sgRNA_Fw/2532 | TATATGAATCGCTTGCCACAATAC | validation CRISPRi |
| glyA_sgRNA_Rv/2533 | AAACGTATTGTGGCAAGCGATTCA | validation CRISPRi |
| sspA_sgRNA_Fw/ 2534 | TATATGAACGTTTACTTGATGCGC | validation CRISPRi |
| sspA_sgRNA_Rv/2535 | AAACGCGCATCAAGTAAACGTTCA | validation CRISPRi |
| mCherry_internal_R_OVL6185/2602 | TGCCATGTTATCTTCTTCTC | CRISPRi library construction |
| mCherry_internal_F_OVL6186/2603 | TGTTGAACAATATGAACGC | CRISPRi library construction |
| sRNAR1_OVL2091/  2604 | TTTTCTCGAGCAAGCAGAAGACGGC | sgRNA distribution |
| P5-N501-read1/2616 | AATGATACGGCGACCACCGAGATCTACACTAGATCGCTCGTCGGCAGCGTCAGATGTGTATA | Illumina sgRNA amplicon library preparation |
| P5-N502-read1/2617 | AATGATACGGCGACCACCGAGATCTACACCTCTCTATTCGTCGGCAGCGTCAGATGTGTATA | Illumina sgRNA amplicon library preparation |
| P5-N503-read1/2628 | AATGATACGGCGACCACCGAGATCTACACTATCCTCTTCGTCGGCAGCGTCAGATGTGTATA | Illumina sgRNA amplicon library preparation |
| P5-N504-read1/2619 | AATGATACGGCGACCACCGAGATCTACACAGAGTAGATCGTCGGCAGCGTCAGATGTGTATA | Illumina sgRNA amplicon library preparation |
| P5-N505-read1/2620 | AATGATACGGCGACCACCGAGATCTACACGTAAGGAGTCGTCGGCAGCGTCAGATGTGTATA | Illumina sgRNA amplicon library preparation |
| P5-N506-read1/2621 | AATGATACGGCGACCACCGAGATCTACACACTGCATATCGTCGGCAGCGTCAGATGTGTATA | Illumina sgRNA amplicon library preparation |
| P5-N507-read1/2622 | AATGATACGGCGACCACCGAGATCTACACAAGGAGTATCGTCGGCAGCGTCAGATGTGTATA | Illumina sgRNA amplicon library preparation |
| P5-N508-read1/2623 | AATGATACGGCGACCACCGAGATCTACACCTAAGCCTTCGTCGGCAGCGTCAGATGTGTATA | Illumina sgRNA amplicon library preparation |
| P7-N701-read2/2624 | CAAGCAGAAGACGGCATACGAGATTCGCCTTAGTCTCGTGGGCTCGGAGATGTGTAT | Illumina sgRNA amplicon library preparation |
| P7-N702-read2/2625 | CAAGCAGAAGACGGCATACGAGATCTAGTACGGTCTCGTGGGCTCGGAGATGTGTAT | Illumina sgRNA amplicon library preparation |
| P7-N703-read2/2626 | CAAGCAGAAGACGGCATACGAGATTTCTGCCTGTCTCGTGGGCTCGGAGATGTGTAT | Illumina sgRNA amplicon library preparation |
| P7-N704-read2/2627 | CAAGCAGAAGACGGCATACGAGATGCTCAGGAGTCTCGTGGGCTCGGAGATGTGTAT | Illumina sgRNA amplicon library preparation |
| P7-N706-read2/2629 | CAAGCAGAAGACGGCATACGAGATCATGCCTAGTCTCGTGGGCTCGGAGATGTGTAT | Illumina sgRNA amplicon library preparation |
| P7-N707-read2/2630 | CAAGCAGAAGACGGCATACGAGATGTAGAGAGGTCTCGTGGGCTCGGAGATGTGTAT | Illumina sgRNA amplicon library preparation |
| P7-N708-read2/2631 | CAAGCAGAAGACGGCATACGAGATCCTCTCTGGTCTCGTGGGCTCGGAGATGTGTAT | Illumina sgRNA amplicon library preparation |
| F_AarI-Hi0601up/2477 | TATTACACCTGCACGTTCCCAAGACGAGTTATCAAAGAATT | CRISPRi –seq validation |
| R_AarI_Hi0601dn/2482 | TATTACACCTGCACGTGGTGACTAAATCTCATCAAAGCAG | CRISPRi –seq validation |
| sgRNA0199-HI_t06-F /2666 | TATAGATTTGAACCGACGGCCTTC | CRISPRi –seq validation |
| sgRNA0199-HI_t06-R /2667 | AAACGAAGGCCGTCGGTTCAAATC | CRISPRi –seq validation |
| sgRNA0351-rpoH-F /2648 | TATATAACCTTCGATACTGCCTTG | CRISPRi –seq validation |
| sgRNA0351-rpoH-R /2649 | AAACCAAGGCAGTATCGAAGGTTA | CRISPRi –seq validation |
| sgRNA3044-dnaE-F /2652 | TATAAAGATGGATGAAGCGAGGTT | CRISPRi –seq validation |
| sgRNA3044-dnaE-R /2653 | AAACAACCTCGCTTCATCCATCTT | CRISPRi –seq validation |
| rplO-sgRNA0895-F /2664 | TATACTATGCTTTGCACCTTCAGC | CRISPRi –seq validation |
| sgRNA0895-rplO-R /2665 | AAACGCTGAAGGTGCAAAGCATAG | CRISPRi –seq validation |
| sgRNA1501-infC-F /2660 | TATAAAACCGGCGGCTCTGCATTT | CRISPRi –seq validation |
| sgRNA1501-infC-R /2661 | AAACAAATGCAGAGCCGCCGGTTT | CRISPRi –seq validation |
| sgRNA1085-dnaA-F /2650 | TATACTTGATCTTGAAGTTGTAAC | CRISPRi –seq validation |
| sgRNA1085-dnaA-R /2651 | AAACGTTACAACTTCAAGATCAAG | CRISPRi –seq validation |
| sgRNA3313-ispE-F/2735 | TATACATTTGATTCTGTTGTATTT | CRISPRi –seq validation |
| sgRNA3313-ispE-R/2736 | AAACAAATACAACAGAATCAAATG | CRISPRi –seq validation |
| sgRNA1235-metK-F /2658 | TATAATTTGATCGGCAATTTTATC | CRISPRi –seq validation |
| sgRNA1235-metK-R /2659 | AAACGATAAAATTGCCGATCAAAT | CRISPRi –seq validation |
| sgRNA0590-rpoC-F /2646 | TATAAATCACATCAAAATCTTCAC | CRISPRi –seq validation |
| sgRNA0590-rpoC-R /2647 | AAACGTGAAGATTTTGATGTGATT | CRISPRi –seq validation |
| sgRNA1129-lpxB-F/2715 | TATACCTGCAACAAGAGCAATGGT | CRISPRi –seq validation |
| sgRNA1129-lpxB-R/2716 | AAACACCATTGCTCTTGTTGCAGG | CRISPRi –seq validation |
| sgRNA1469-parE-F/  2725 | TATAAATTTCTTGAGCTGAATAAT | CRISPRi –seq validation |
| sgRNA1469-parE-R  /2726 | AAACATTATTCAGCTCAAGAAATT | CRISPRi –seq validation |
| sgRNA1802-rsxA-F/2719 | TATATACAAAATTGTTAATTAATG | CRISPRi –seq validation |
| sgRNA1802-rsxA-R/2720 | AAACCATTAATTAACAATTTTGTA | CRISPRi –seq validation |
| sgRNA0304-guaB-F/2709 | TATATAGAACATCGTCAAAAGTAA | CRISPRi –seq validation |
| sgRNA0304-guaB-R/2710 | AAACTTACTTTTGACGATGTTCTA | CRISPRi –seq validation |
| sgRNA1253-ilvE-F/2703 | TATATTTTAATATAACTAAAACCA | CRISPRi –seq validation |
| sgRNA1253-ilvE-R/2704 | AAACTGGTTTTAGTTATATTAAAA | CRISPRi –seq validation |
| sgRNA2978-serA-F/2727 | TATATATTGGTATAGCCTGCCGCA | CRISPRi –seq validation |
| sgRNA2978-serA-R/2728 | AAACTGCGGCAGGCTATACCAATA | CRISPRi –seq validation |
| sgRNA1873-rpsT-F/2656 | TATAGTTGGCTTGCGTTATGTTGG | CRISPRi –seq validation |
| sgRNA1873-rpsT-R/2657 | AAACCCAACATAACGCAAGCCAAC | CRISPRi –seq validation |
| sgRNA2058-mepA-F/2662 | TATATACCATCTTCACTTGGAATA | CRISPRi –seq validation |
| sgRNA2058-mepA-R/2663 | AAACTATTCCAAGTGAAGATGGTA | CRISPRi –seq validation |
| sgRNA1207-ftsZ-F/2767 | TATATATCGTACTCAGGGTACTCT | CRISPRi –seq validation |
| sgRNA1207-ftsZ-R/2768 | AAACAGAGTACCCTGAGTACGATA | CRISPRi –seq validation |
| sgRNA0695-rpsL-F/2723 | TATACACGCGGTTTGCGTACTAGC | CRISPRi –seq validation |
| sgRNA0695-rpsL-R/2724 | AAACGCTAGTACGCAAACCGCGTG | CRISPRi –seq validation |
| f1_*serA*_new/2333 | CAAATATGACAAACAAAGTTTCACTCG | ∆*serA* disruption cassette |
| r1_*serA*_new/2334 | TAGTAAAGTACTCTAGCACGAATTGTG | ∆*serA* disruption cassette |
| *serA*_F2_NEW/2376 | TTCTTGCGATGTGGTTTCATTACATGT | ∆*serA* disruption cassette |
| *serA*_R2_NEW/2377 | taattcttcaaggctacgaacctgttt | ∆*serA* disruption cassette |


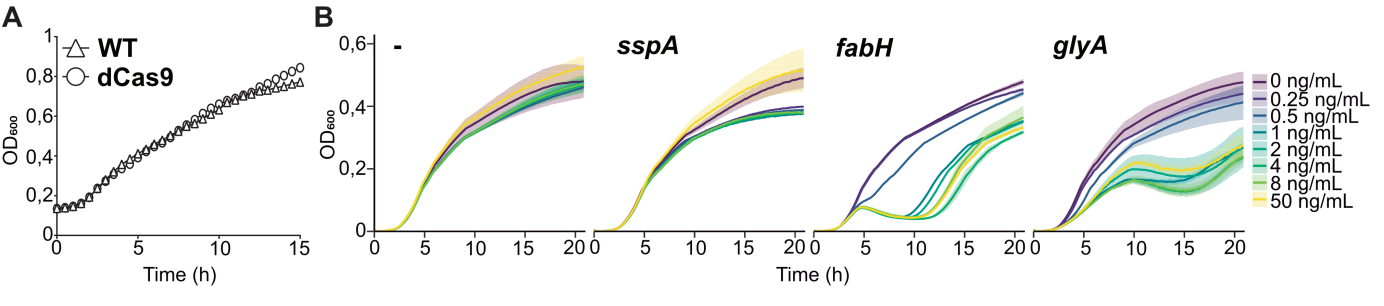


**Fig A. Dynamic range of the aTc-inducible CRISPRi system in *H. influenzae*.**

**(A)** Growth of *H. influenzae* WT (triangles) and *dcas9* (circles) strains in sBHI. **(B)** P_tet_ responsiveness experiment: *dcas9* strains carrying no (-) or sgRNAs targeting the *sspA*, *fabH* or *glyA* genes were grown for 12 h at 37°C in 10 mL sBHI with shaking (120 r.p.m). Next, cultures were diluted in fresh sBHI to OD_600_=0.01 and transferred to a 96-wells plate with a range of aTc concentrations from 0.25 to 50 ng/mL. The color-coded shadow area represents the standard deviation to the mean (solid line).


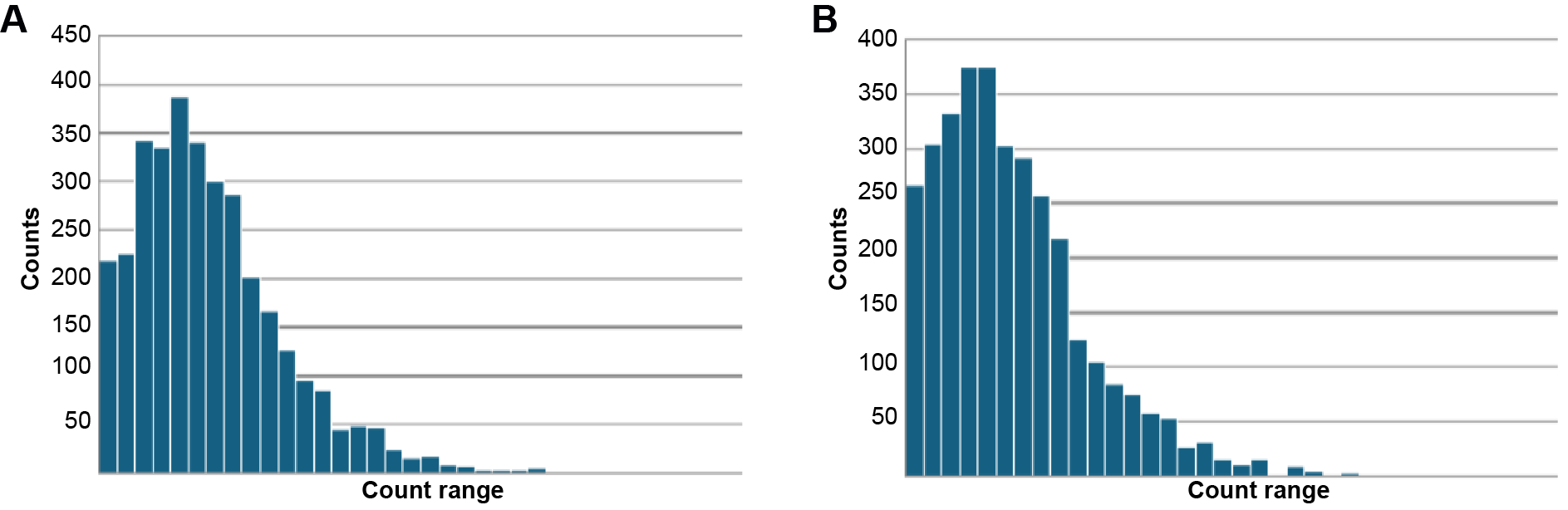

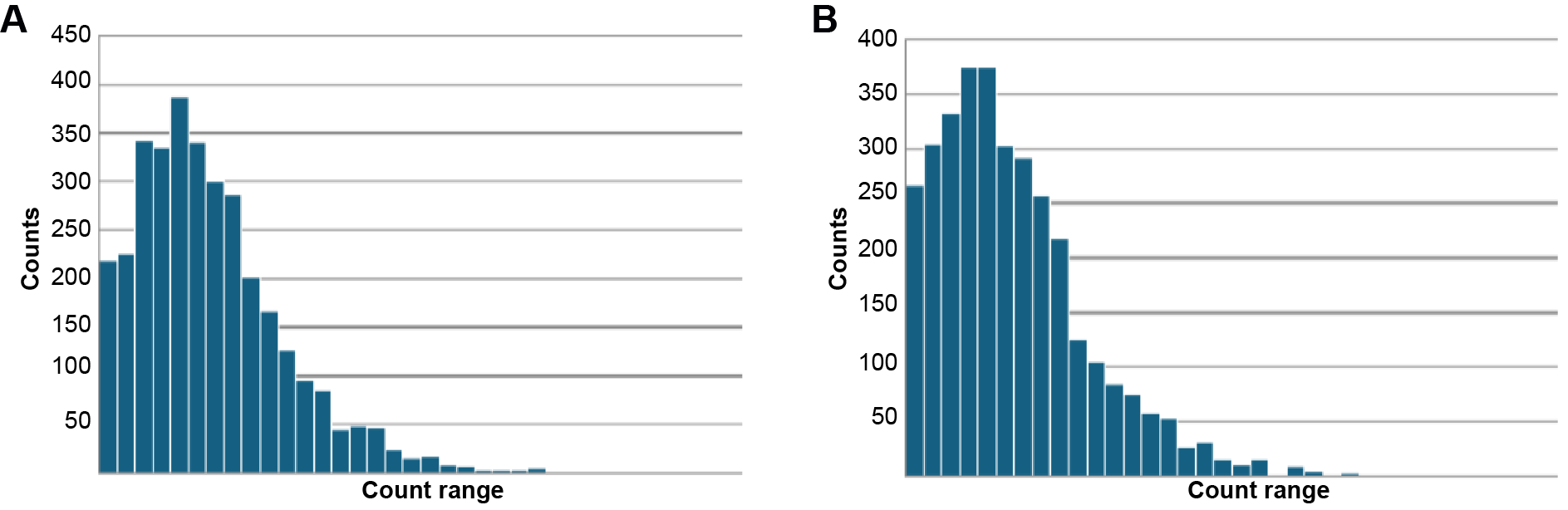
**
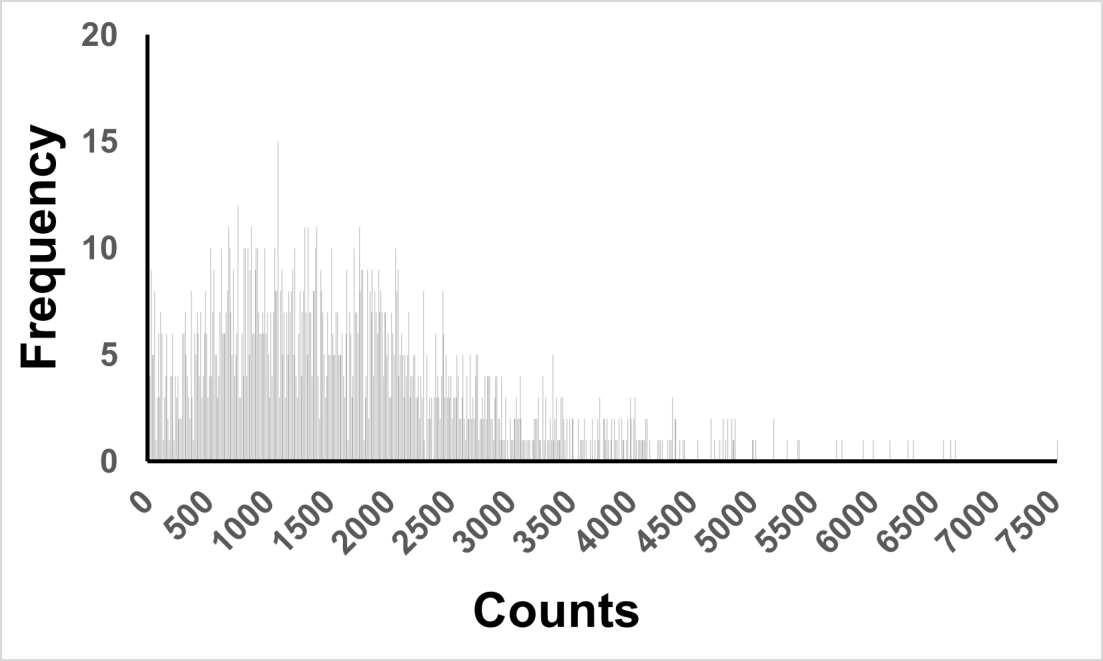

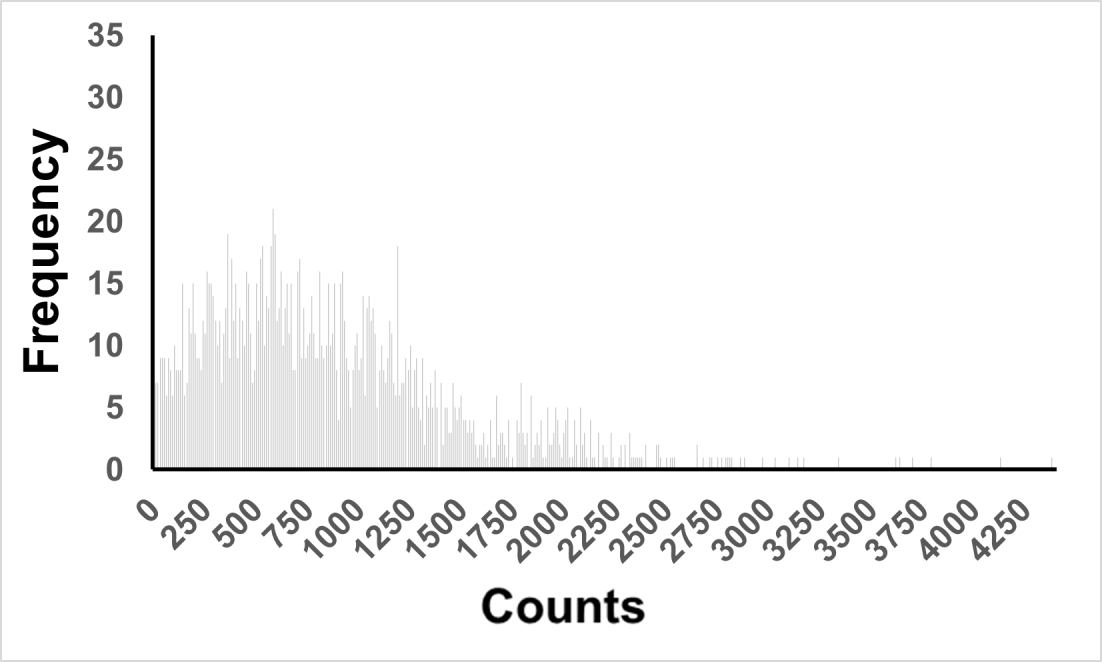
**

**Fig B.** **Frequency histogram of the *E. coli* and *H. influenzae* sgRNA library distribution.**

sgRNA library distribution in *E. coli* **(A)** and in *H. influenzae* **(B)**.


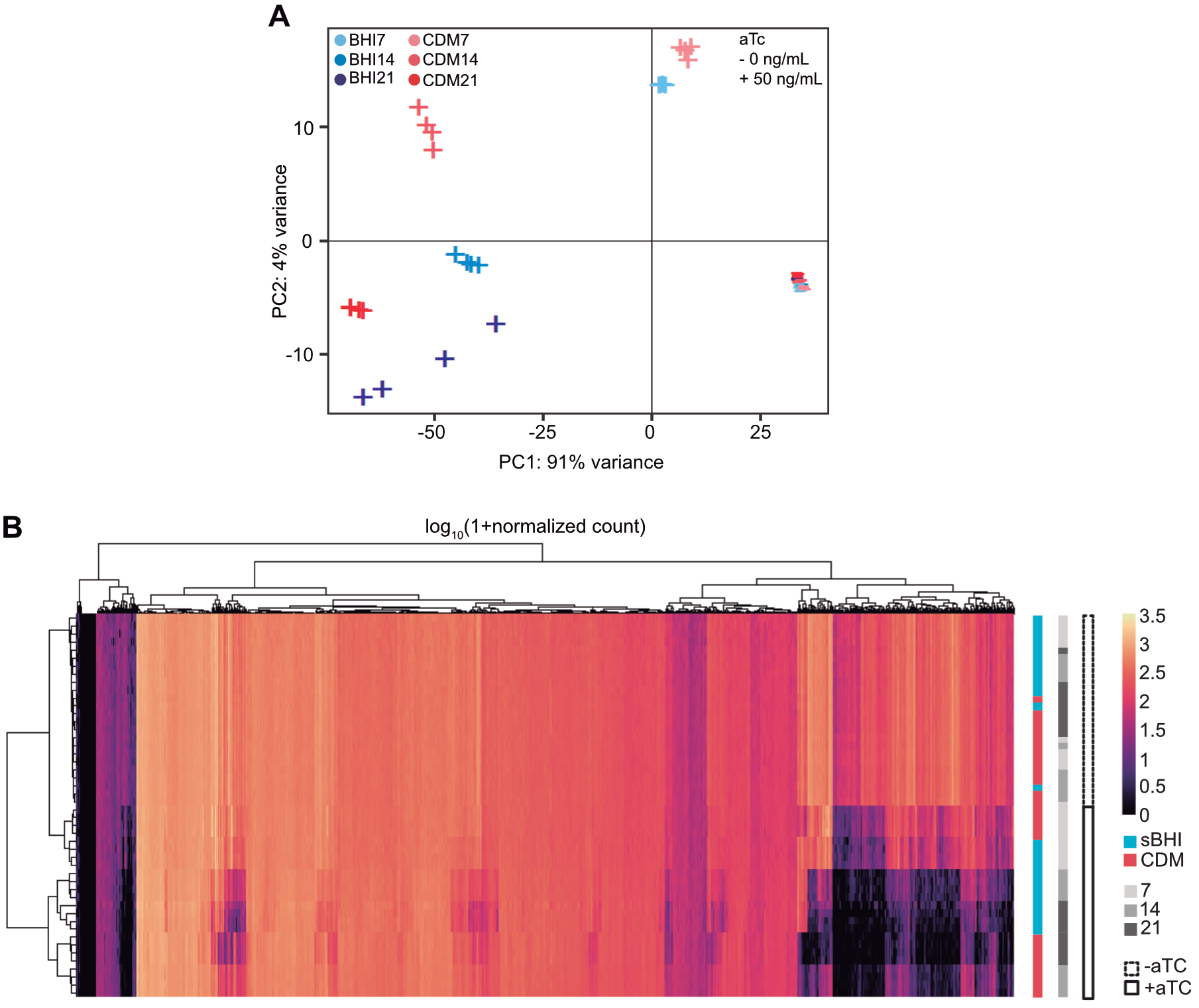


**Fig C. sgRNA content variation during CRISPRi screen in sBHI and CDM.**

**(A)** Principal component analysis (PCA) shows the disparity between replicates, induction (+ or – aTc), media (sBHI, CDM) and timepoints (7, 14 and 21 generations). + signs represent samples where CRISPRi was activated by aTc; - signs show samples where CRISPRi was uninduced. Blue and red signs represent CRISPRi libraries grown in sBHI and CDM, respectively. Increasing the induction time explains most of the variance in sample strain composition (91%). **(B)** Clustered heatmap of sgRNA content between replicates, induction (+ or – aTc), media (sBHI, CDM) and timepoints (7, 14 and 21 generations). The normalized read count is expressed in a gradient logarithmic scale. The horizontal axis displays the 1,773 sgRNAs designed for the RdKW20 CRISPRi library, while the vertical axis shows the 48 different samples.

**
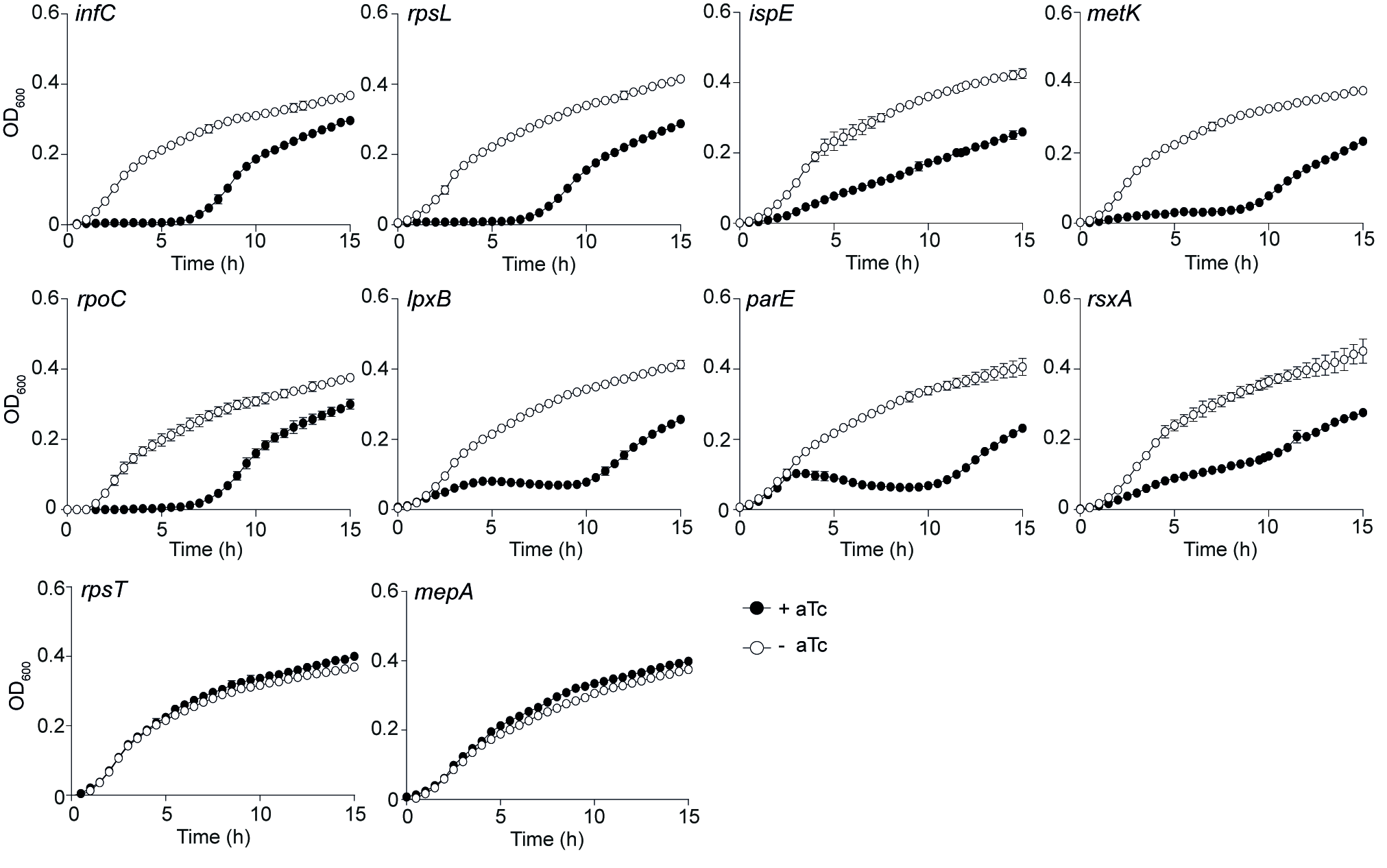
**

**Fig D. CRISPRi-based analysis of *H. influenzae* specific gene fitness in sBHI.**

*dcas9* derivative strains expressing *infC*, *rpsL*, *ispE*, *metK*, *rpoC*, *lpxB*, *parE, rsxA*, *rpsT* and *mepA* sgRNAs were grown in sBHI, in the absence (white circles)/presence (black circles) of aTc. Strains were grown in 96-well plates, OD_600_ was measured every 30 min for 15 h; standard deviation to the mean is shown for each timepoint.

**
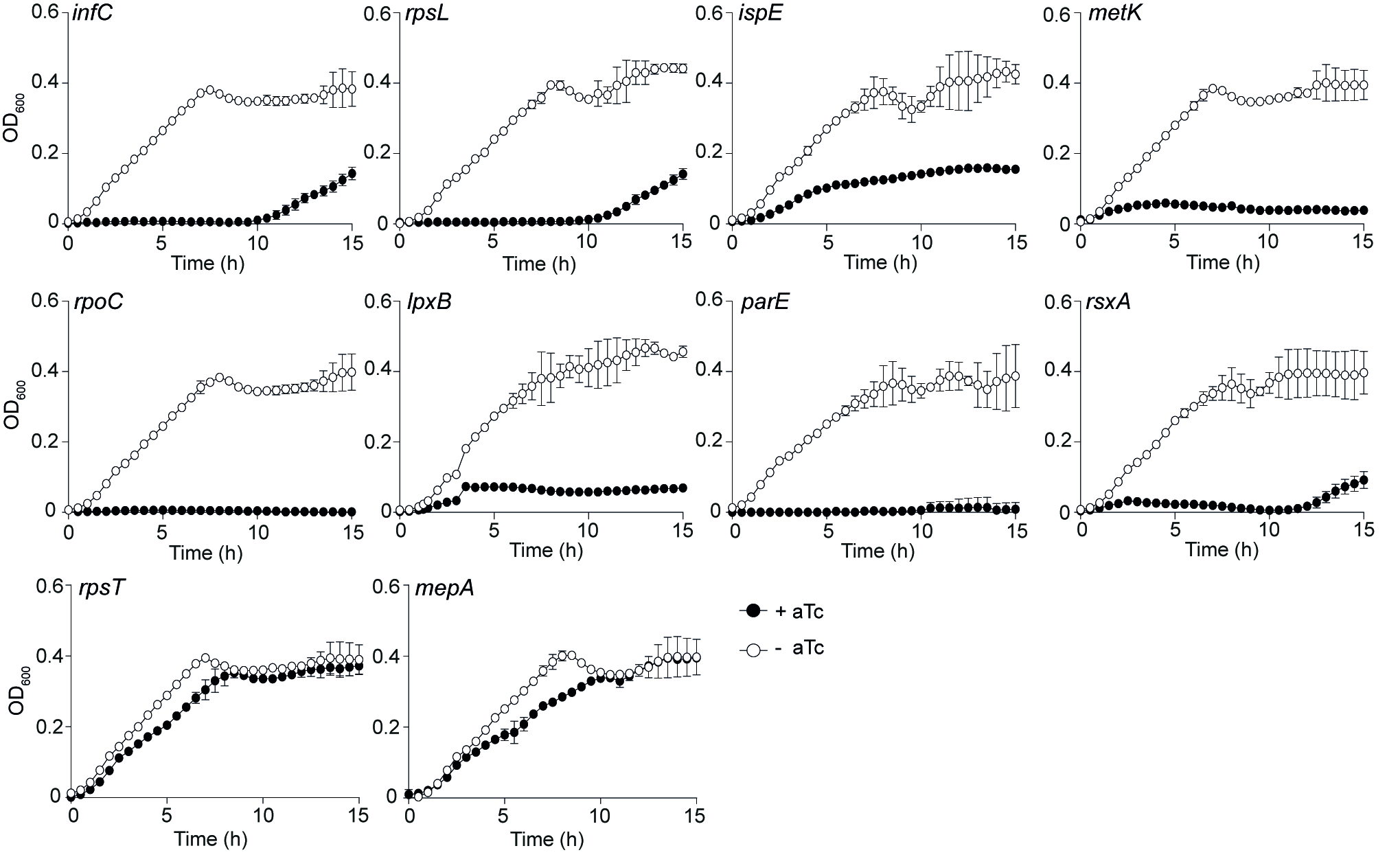
**

**Fig E. CRISPRi-based analysis of *H. influenzae* specific gene fitness in CDM.**

*dcas9* derivative strains expressing *infC*, *rpsL*, *ispE*, *metK*, *rpoC*, *lpxB*, *parE,* and *rsxA*, *rpsT* and *mepA* sgRNAs were grown in CDM, in the absence (white circles)/presence (black circles) of aTc. Strains were grown in 96-well plates, OD_600_ was measured every 30 min for 15 h; standard deviation to the mean is shown for each timepoint.


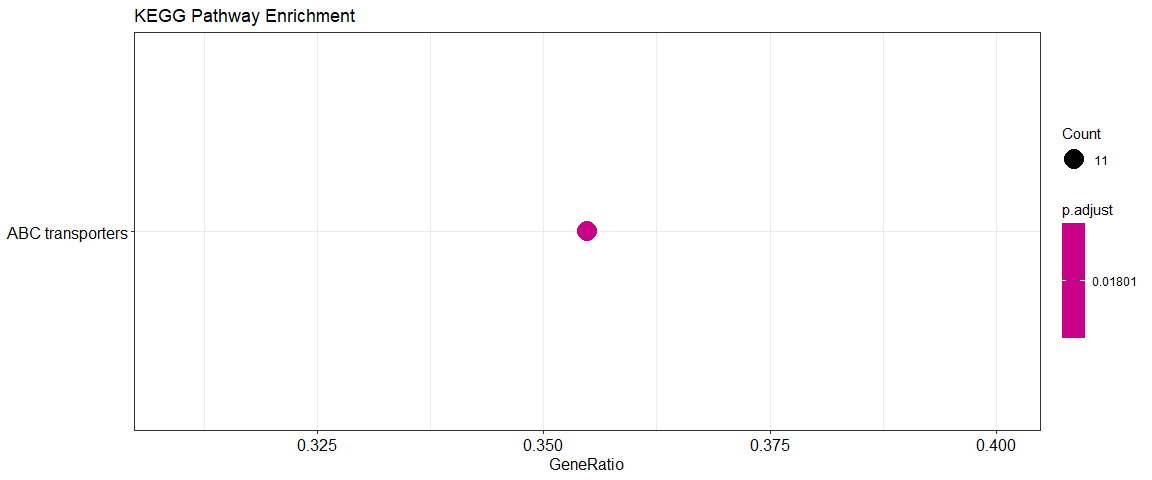


**Fig F. Pathway enrichment in sBHI.**

KEGG pathway enrichment analysis in sBHI (versus CDM). The gene ratio refers to the number of enriched genes in sBHI for a specific pathway reported to the total amount of differentially essential genes. Dot size reflects the number of differentially essential genes. Color shades scale the significance (Padj).


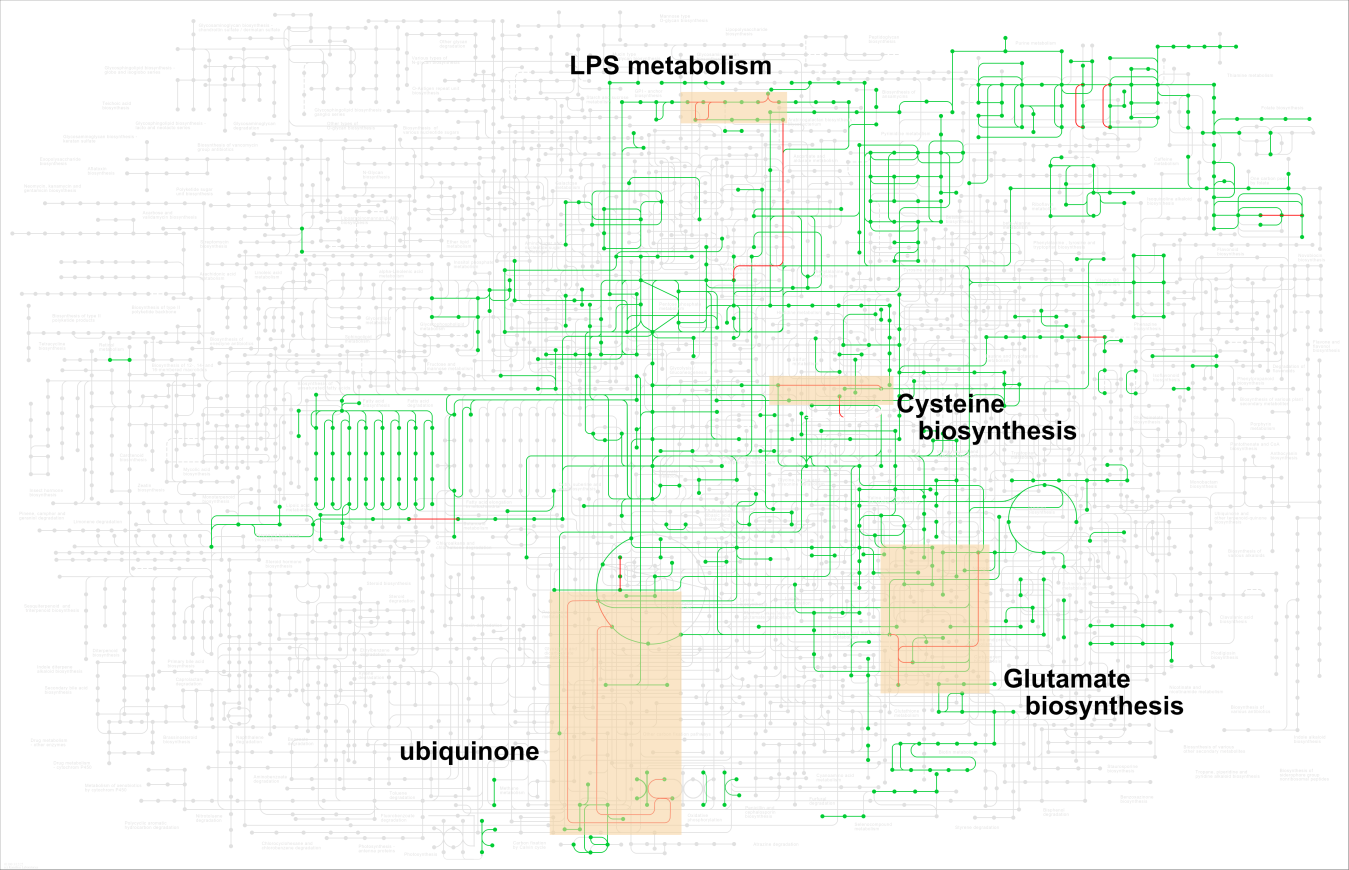


**Fig G. Metabolic map of differentially essential gene enrichment in sBHI.**

KEGG map (hin01100) of the metabolic landscape in *H. influenzae* RdKW20 (green). The nodes represent metabolic compounds, and edges represent genes involved in the reactions. Pathways with enrichment in differentially essential genes are framed in Orange. Metabolic enzymes differentially more essential in sBHI (*versus* CDM) are highlighted in red.


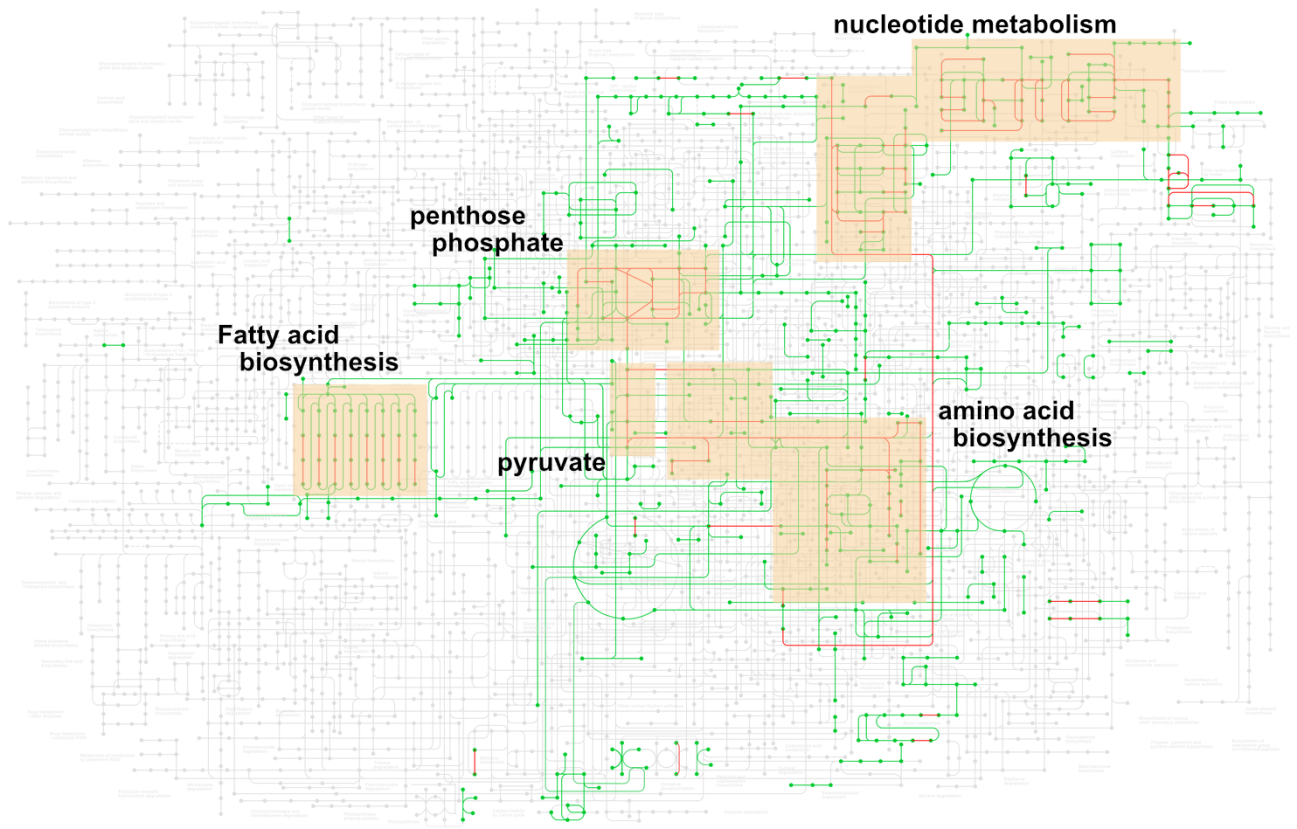


**Fig H. Metabolic map of differentially essential gene enrichment in CDM.**

KEGG map of the metabolic landscape in *H. influenzae* RdKW20 (green). The nodes represent metabolic compounds and edges represent genes involved in the reactions. Pathways with enrichment in differentially essential genes are framed in Orange. Metabolic enzymes differentially more essential in CDM (*versus* sBHI) are highlighted in red.


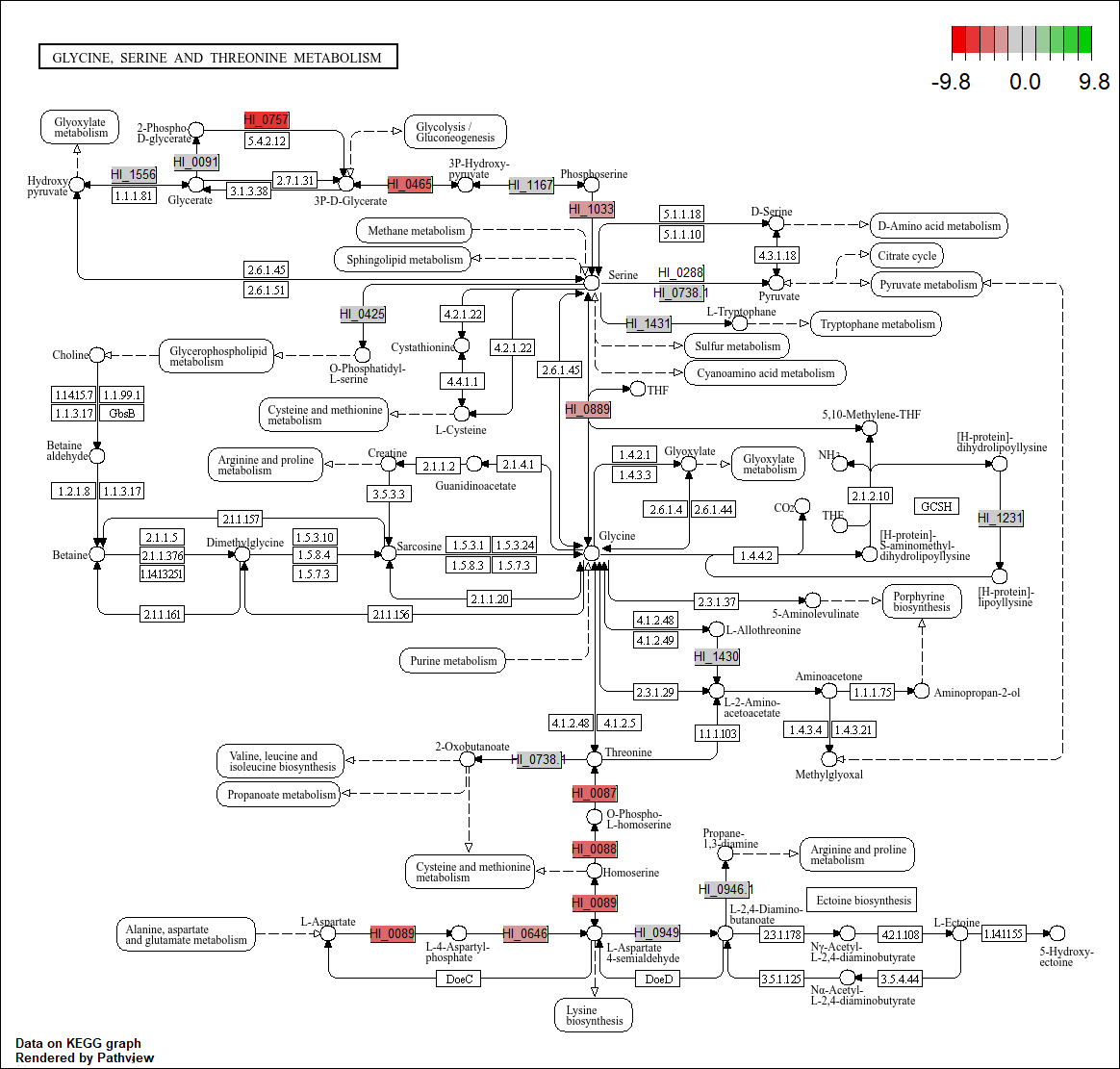


**Fig I.** **Differentially essential genes in the glycine, serine and threonine pathway.**

Path view mapper visualization of enriched/depleted genes using the KEGG database (hin00260) in CDM *versus* sBHI for the glycine, serine and threonine metabolism. KEGG reference pathway numbers are replaced by gene names when homologs are encoded in the RdKW20 genome. The log_2_FC of fitness scores (from DEseq2 analysis) are depicted with a gradient color scale. Red color indicates a negative log_2_FC.

**
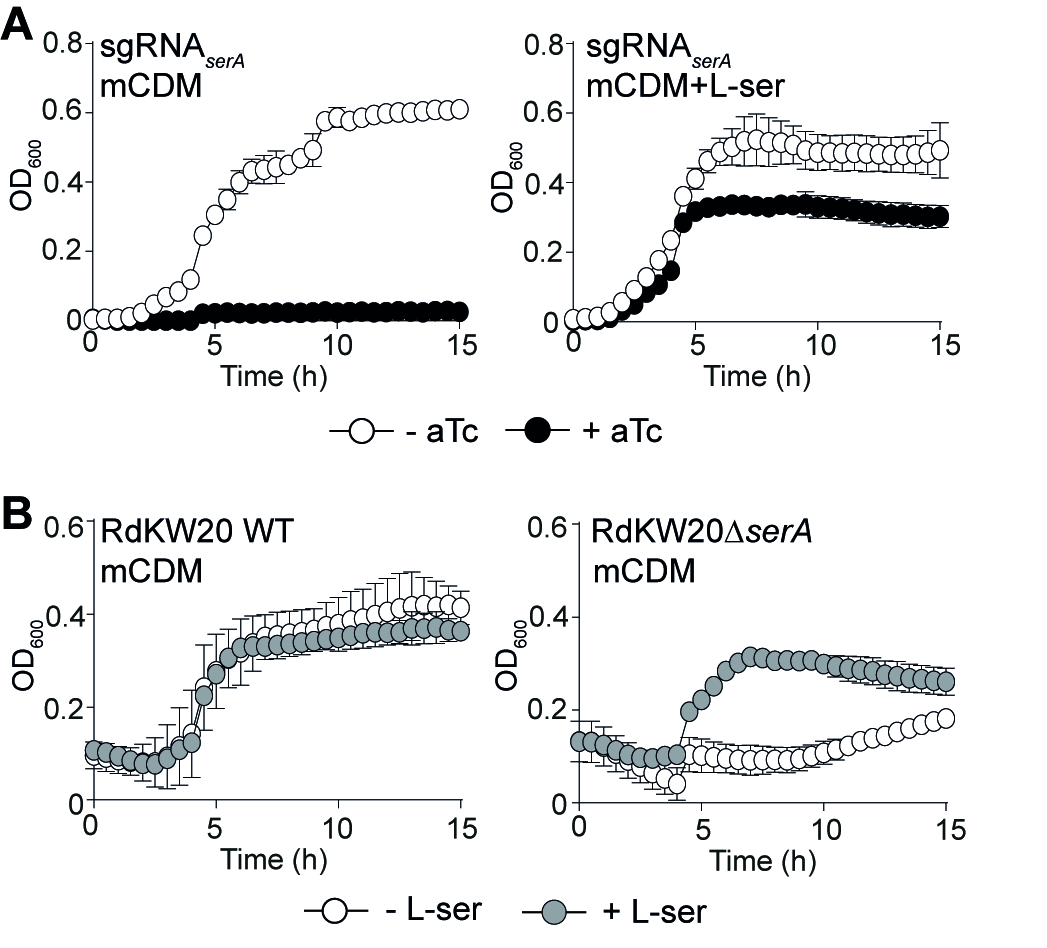
**

**Fig J.** **sgRNA gene silencing reversibility in *H. influenzae*.**

**(A)** The*dcas9* sgRNA*_serA_* strain was grown in mCDM (left) or mCDM+L-serine 10 mM (right), in the absence (white circles)/presence (black circles) of aTc. **(B)** RdKW20 WT and Δ*serA* mutant strains were grown in mCDM in the absence (white circles)/presence (grey circles) of L-serine 10 mM.

**References**

1. Fleischmann RD, Adams MD, White O, Clayton RA, Kirkness EF, Kerlavage AR, Bult CJ, Tomb JF, Dougherty BA, Merrick JM, McKenney K, Sutton G, FitzHugh W, Fields C, Gocayne JD, Scott J, Shirley R, Liu LI, Glodek A, Kelley JM, Weidman JF, Phillips CA, Spriggs T, Hedblom E, Cotton MD, Utterback TR, Hanna MC, Nguyen DT, Saudek DM, Brandon RC, Fine LD, Fritchman JL, Fuhrmann JL, Geoghagen NSM, Gnehm CL, McDonald LA, Small K V., Fraser CM, Smith HO, Venter JC. 1995. Whole-genome random sequencing and assembly of *Haemophilus influenzae* Rd. Science (80- ) 269:496–512.

2. Mell JC, Sinha S, Balashov S, Viadas C, Grassa CJ, Ehrlich GD, Nislow C, Redfield RJ, Garmendia J. 2014. Complete genome sequence of *Haemophilus influenzae* strain 375 from the middle ear of a pediatric patient with otitis media. Genome Announc 2(6):e01245-14.

3. Harrison A, Dyer DW, Gillaspy A, Ray WC, Mungur R, Carson MB, Zhong H, Gipson J, Gipson M, Johnson LS, Lewis L, Bakaletz LO, Munson Jr. RS. 2005. Genomic sequence of an otitis media isolate of nontypeable *Haemophilus influenzae*: comparative study with H. influenzae serotype d, strain KW20. J Bacteriol 187:4627–4636.

4. Reilly TJ, Chance DL, Smith AL. 1999. Outer membrane lipoprotein e (P4) of *Haemophilus influenzae* is a novel phosphomonoesterase. J Bacteriol 181:6797–6805.

5. Othman DSMP, Schirra H, McEwan AG, Kappler U. 2014. Metabolic versatility in *Haemophilus influenzae*: A metabolomic and genomic analysis. Front Microbiol 5:1–10.

6. López-López N, Euba B, Hill J, Dhouib R, Caballero L, Leiva J, Hosmer J, Cuesta S, Ramos-Vivas J, Díez-Martínez R, Schirra HJ, Blank LM, Kappler U, Garmendia J. 2020. *Haemophilus influenzae* glucose catabolism leading to production of the immunometabolite acetate has a key contribution to the host airway-pathogen interplay. ACS Infect Dis 6:406–421.

7. de Bakker V, Liu X, Bravo AM, Veening J-W. 2022. CRISPRi-seq for genome-wide fitness quantification in bacteria. Nat Protoc 17:252–281.
