# Supplementary material for: CRISPRi-seq in Haemophilus influenzae reveals genome-wide and medium-specific growth determinants
Source: PLoS Pathog. 2025 Oct 31;21(10):e1013650. doi: 10.1371/journal.ppat.1013650 (PMC12591398; doi:10.1371/journal.ppat.1013650)
Supplement: Attachment — Submitted filename: 00_Response to reviewers.docx [file ppat.1013650.s009.docx]

**RESPONSE TO REVIEWERS**

Dear Editors,

We appreciate very much the revision and constructive comments on our manuscript **PPATHOGENS-D-25-01952 “CRISPRi-seq in *Haemophilus influenzae* reveals genome-wide and medium-specific growth determinants”**.

We understand the need for the requested modifications. Following all indications made by the three reviewers, we have modified the main text to fulfill all requests.

Moreover, we have answered to the best of our capacity all the issues raised by the three reviewers. Please find next a point-by-point answer to all the comments, and find attached the revised Main text, Supplemental Material and Figures.

Sincerely,

Junkal Garmendia and Jan-Willem Veening

**Journal Requirements:**

1) We do not publish any copyright or trademark symbols that usually accompany proprietary names, eg ©,  ®, or TM  (e.g. next to drug or reagent names). Therefore please remove all instances of trademark/copyright symbols throughout the text, including:

- ® on page: 23

- TM on page: 21.

These symbols have been removed.

2) We have noticed that you have uploaded Supporting Information files, but you have not included a list of legends. Please add a full list of legends for your Supporting Information files after the references list.

As requested, a list of legends for Supporting Datasets, Tables and Figures is now included after the reference list.

3) Some material included in your submission may be copyrighted.

Please respond directly to this email and provide any known details concerning your material's license terms and permissions required for reuse, even if you have not yet obtained copyright permissions or are unsure of your material's copyright compatibility. Once you have responded and addressed all other outstanding technical requirements, you may resubmit your manuscript within Editorial Manager.

 Potential Copyright Issues:

i) Figures 1C, and 2A. Please confirm whether you drew the images / clip-art within the figure panels by hand.

We confirm that Figures 1 and 3 have been modified, and revised versions of both figures have been handdrawn by ourselves.

ii) The following Figure contains screenshots: 7D. We are not permitted to publish these under our CC-BY 4.0 license, websites are usually intellectual property and are copyrighted. This includes peripheral graphics of the web browser such as icons and button. We ask that you please remove or replace it.

Panel 7D depicts data generated by- and available through AlphaFold. In previous work, we published genome browsers with similar figures (mBio; <https://journals.asm.org/doi/pdf/10.1128/mbio.00840-24>, and NAR; <https://academic.oup.com/nar/article/53/D1/D839/7831090>). In these studies, we have included a reference to the AlphaFold work, as well as other external resources. We have now referenced the appropriate works in the main text (results, methods and figure legend texts), and hope that this additional attribution of these published works satisfies this point.

As the HaemoBrowse resource is an important aspect of this manuscript, as highlighted by the referees, we are of the opinion that showing the availability of cross-referenced databases is a key aspect of this work. As the referenced works constitute academic contributions to scientific literature and are freely available from the web, the referencing of the applicable works should satisfy the conditions that are necessary for publishing.

4) Thank you for stating "Data available as a BioProject, ID: PRJNA1274682." Please note that, though access restrictions are acceptable now, your entire minimal dataset will need to be made freely accessible if your manuscript is accepted for publication. This policy applies to all data except where public deposition would breach compliance with the protocol approved by your research ethics board.

The date of release for the BioProject, ID: PRJNA1274682." had been brought forward to the 30th of October 2025 or upon publication, whichever is first.

5) Thank you for indicating that “HaemoBrowse is freely available at [https://HaemoBrowse.VeeningLab.com](https://haemobrowse.veeninglab.com/).” Thank you for uploading your study's underlying data set. Unfortunately, the repository you have noted in your Data Availability statement does not qualify as an acceptable data repository according to PLOS's standards. At this time, please upload the minimal data set necessary to replicate your study's findings to a stable, public repository (such as figshare or Dryad) and provide us with the relevant URLs, DOIs, or accession numbers that may be used to access these data. For a list of recommended repositories and additional information on PLOS standards for data deposition, please see

<https://journals.plos.org/plospathogens/s/recommended-repositories>

Thank you for this comment. Please note that the HaemoBrowse resource has not been designed to replace public repositories of published data. Our goal was to make the data included in this paper more accessible through a visual browser. All the underlying data in HaemoBrowse is present in the (supplementary) tables-datasets associated with this paper, or through the described methods.

6) Please amend your detailed Financial Disclosure statement. This is published with the article. It must therefore be completed in full sentences and contain the exact wording you wish to be published.
1) State what role the funders took in the study. If the funders had no role in your study, please state: "The funders had no role in study design, data collection and analysis, decision to publish, or preparation of the manuscript."

This modification has been introduced in “Funding” paragraph: The funders had no role in study design, data collection and analysis, decision to publish, or preparation of the manuscript.

7) Thank you for stating "." Please declare all competing interests beginning with the statement "I have read the journal's policy and the authors of this manuscript have the following competing interests:"

This modification has been introduced: "I have read the journal's policy and the authors of this manuscript have the following competing interests: J.W.V. is a scientific advisory board member at i-Seq Biotechnology.

8) Please ensure that the funders and grant numbers match between the Financial Disclosure field and the Funding Information tab in your submission form. Note that the funders must be provided in the same order in both places as well.

All grants and funds have been reported in the funding paragraph. Funders are provided in the same order in both places.

**Reviewers' Comments:**

**Part I – Summary.** Please use this section to discuss strengths/weaknesses of study, novelty/significance, general execution and scholarship.

**Reviewer #1:** This paper presents a fascinating development in functional genomics to define essential genes and explore fitness impacts across the entire H. influenzae genome. There are current limitations (most notably use of model strain Rd which is not fully representative of either encapsulated or nonencapsulated lineages in carriage or disease), and the conditions for which fitness is tested are fairly limited...reviewer would certainly like to see this work extended into more relevant strains/lineages and conditions associated with carriage or virulence. These points certainly don't limit the significance or impact of the work in this paper.

Thank you for this comment, it is very much appreciated. Certainly, this study presents a new genome-wide platform for fitness studies that we hope to be useful to move forward our understanding on *H. influenzae* fitness within the host. As we indicate in the revised discussion section, our current observations **open a whole range of clinically relevant *in vitro* or *in vivo* conditions to be investigated in future studies.**

**Also, additional text is now included in the discussion section to clarify that translation of our CRISPRi platform to other strains of clinical relevance in different anatomical niches will be also investigated. Indeed, this important issue was part of our study design given that, as indicated in the Methods section, our sgRNA library was design to target every genetic feature of the** RdKW20, NTHi375, 86-028NP and R2866 strains, and tested on the RdKW20 CRISPRi newly generated platform.

**Reviewer #2:** This manuscript by Gil-Campillo et al. describes the implementation and validation of an inducible CRISPR system for mutant generation in Haemophilus influenzae. The manuscript is well written and presented in a clear, logical manner that is well supported by the data. The approach is sound and strengthened by the use of varied methodology and phenotypic complementation. While the CRISPRi approach has been described in other bacteria, there is novelty in its application to Haemophilus as well as in the development of the HaemoBrowse database generated in this work. The validation of identified genes through direct mutagenesis, complementation, and phenotypic assays is a significant strength of the study.

Thank you for these comments, they are very much appreciated.

**Reviewer #3:** This is a commendable study in which the authors report a comprehensive functional genomics tool for H. influenzae strain Rd KW20. While primarily a methods paper, the authors demonstrate cell and physiological phenotypes, such as cell division and nutrient utilization, that can be studied using the sgRNA conditional expression system. Overall, the findings are straightforward and confirm or extend results of previous essential gene identifications provided by transposon insertion site sequencing studies and demonstrate utility of an inducible genetic repression system that allows detailed confirmation and follow-up functional analyses of genes involved in fitness under varied conditions.

Thank you for these comments, they are very much appreciated.

It would be useful to know the approximate percentage of sgRNA constructs that are functionally able to deplete their target RNAs.

As stated in de Bakker at al. (Nat Protoc. 2022 Feb;17(2):252-281. doi: 10.1038/s41596-021-00639-6), every sgRNAs have been designed/optimized to obstruct transcript elongation at early stages, and maximize the repression activity (non-template strand target and close proximity to the primary operon TSS or start codon). To evaluate the actual percentage of functional sgRNAs and accurately quantify depletion rate for every single gene, single-cell RNA-seq would be the only mean, which we consider out of the scope of this study.

For completion, we have compared our CRISPRi-seq data with previously published studies where transposon mutagenesis was used and gene essentiality elucidated. For this purpose, we have compared RdKW20 essential genes upon sBHI growth *in vitro* (Mobegi et al., 2014, BMC Genomics. doi: 10.1186/1471-2164-15-958) and *in vivo*, i.e. after passing through a mouse intranasal infection model (Garownski et al., Proc Natl Acad Sci U S A. 2009 Sep 22;106(38):16422-7. doi: 10.1073/pnas.0906627106; Gil-Campillo et al., mBio. 2025 Aug 13;16(8):e0135525. doi: 10.1128/mbio.01355-25.). Indeed, 262 genes were found to be essential after both CRISPRi-seq and Tn-seq screening, and 340 genes were found to be essential by CRISPRi-seq but not by Tn-seq; however, only 47 genes were found to be essential in Tn-seq but not in CRISPRi-seq screenings (see **newly added Dataset S6**). We consider that, altogether and considering intrinsic differences among studies, such comparisons further support the reliability of the approach presented in this study.

From the standpoint of studying H. influenzae as a pathogen, a missed opportunity was the lack of an animal infection study, as the tool could potentially clarify which of the *in vitro* essential genes are also essential for infection.

Thank you for this comment. We agree that using our CRISPRi-seq platform in more physiological model systems will add very valuable information in terms of bacterial genes essential for infection, also unravelling those with potential druggable targets.

Indeed, please note that the previously developed CRISPRi-seq platform for *Streptococcus pneumoniae* has been successfully used for *in vivo* screening of bacterial genes required for fitness during mouse infection in previous works (<https://pubmed.ncbi.nlm.nih.gov/38417443/>; <https://pubmed.ncbi.nlm.nih.gov/33120116/>).

Regarding the use of *H. influenzae in vivo* models of infection for gene essentiality studies, several Tn-seq studies have generated information in this regard (please see
**newly added Dataset S6).** We consider that use of our CRISPRi-seq platform to screen bacterial fitness *in vivo* exceed the scope of this study. We agree that this is highly relevant point, and we plan to assess it in future work, as stated in the Discussion section.

Line 180: The term 'genetic features' is vague. Describe the type of elements that were targeted by the design. (eg. all protein coding genes, RNA genes, +?).

By genetic features, we mean all prokka-annotated coding sequences, transfer RNAs, ribosomal RNAs and small RNAs. We modified the text to clarify this point.

Lines 319-322: Some studies conducting transposon-based essential gene identification in H. influenzae were omitted and could improve the comparison. The comparison of the identified essential gene set with those generated in transposon studies should be reported directly as a list, with some discussion of genes that were discordant between methods. Also, were there any genes identified that were not found in transposon-based studies?

Thank you for this comment.

As stated above, we have compared our CRISPRi-seq data with previously published studies where transposon mutagenesis was used and gene essentiality elucidated. In particular, we have compared RdKW20 essential genes upon sBHI growth *in vitro* (Mobegi et al., 2014, BMC Genomics. doi: 10.1186/1471-2164-15-958) and *in vivo*, i.e. after passing through a mouse intranasal infection model (Garownski et al., Proc Natl Acad Sci U S A. 2009 Sep 22;106(38):16422-7. doi: 10.1073/pnas.0906627106; Gil-Campillo et al., mBio. 2025 Aug 13;16(8):e0135525. doi: 10.1128/mbio.01355-25.). Indeed, 262 genes were found to be essential after both CRISPRi-seq and Tn-seq screening, 340 genes were found to be essential by CRISPRi-seq but not by Tn-seq, and only 47 genes found to be essential in Tn-seq but not in CRISPRi-seq screenings (see **newly added Dataset S6**). These comparisons are now indicated in the discussion section as requested.

Lines 337-338: This sentence confounds an experimental caveat (mixed competition) with statistical significance.

Thank you for this comment. The text has been modified to avoid confusion.

Line 348: The term "showcased medium specificities" is ambiguous. Do the authors mean differences in media composition? If so, this is circular logic as we already know the media compositions are different, and the main constituents are known, so it is not clear why it would need to be showcased.

Thank you for this comment. The text has been modified aiming to avoid confusion.

Lines 355-359: The phrase "differentially essential gene enrichment in sBHI" should be clearly defined. The specific genes under discussion should be named and citations provided for the functions ascribed to these genes. The data supporting this discussion should be included in the manuscript so that readers do not need to access the online portal to interpret the statements.

Thank you for this comment. The text has been modified accordingly to provide specific gene information as requested.

Line 356: The authors should comment on whether the insertion of all of the sgRNA constructs into the xylose locus may have influenced essentiality results seen with xylose transport genes.

We designed the *dcas9* cassette insertion to have no or minimal impact on the neighbour genes. First, the *xylB* gene is upstream of and in the same orientation than the *dcas9* cassette. Second, the *dcas9* cassette is bordered by transcriptional terminators to minimize transcriptional readthrought. Third, neither deletion nor duplication were generated due to the *dcas9* cassette insertion. We clarified this point in the first Results section.

**Part II – Major Issues: Key Experiments Required for Acceptance**

**Reviewer #1:**

As noted, ideally this would be extended to relevant strains or conditions, including in vitro or in vivo infection models. I think it's fair for that to be mentioned in discussion as an important future goal.

Thank you for this comment. As stated above, the discussion has been modified to include this aspect.

**Reviewer #2:** None

**Reviewer #3:** (No Response)

**Part III – Minor Issues: Editorial and Data Presentation Modifications**

**Reviewer #1:** No Concerns

**Reviewer #2:**

For many of the induced mutants, outgrowth is observed around 8–10 hours under several assay/growth conditions. This observation should be more directly addressed in the Discussion. Is this due to loss of induction or the emergence of suppressor mutants? Some discussion or a working hypothesis should be provided.

We observed this phenomenon in multiple cases involving different bacterial clades ([doi.org/10.1101/2024.08.14.607739](https://doi.org/10.1101/2024.08.14.607739); doi.org/10.1038/s41467-025-61814-x).

We suspect multifactorial effects to explain this regrow behaviour. First, aTc is a light sensitive compound that will rapidly degrade over time when supplemented in the growth medium. De-repression of *dcas9* can be alleviated at late stages (around 10 h) of the growth culture, allowing target gene expression. Second, considering essential genes, mutations in the *tetR* (repressor) gene or in the *tet* promoter will outcompete cells that fully repress the target gene. Third, we cannot rule out partial repressions for which proteins slightly accumulate in the cytoplasm over time to reach a level that promotes a net growth. Given that the screen was performed over maximum 21 generations (approximately 10 h), this regrowth effect will have a minor impact on data analysis output. We clarified this point in the Discussion section.

Throughout, “MI-V” should be changed to “M-IV” for consistency with the original cited publication.

Thank you for this comment, the modification has been made as requested.

Line 296: remove the repeated word “designed”

Changed.

**Reviewer #3:** (No Response)
